# Validation of two swabbing methods to sample DNA for genotyping Atlantic bluefin tuna (*Thunnus thynnus)*

**DOI:** 10.1038/s41598-025-87053-0

**Published:** 2025-02-11

**Authors:** D. Righton, F. Garzon, L. A. Hawkes, R. Hicks, T. Horton, M. Ives, I. Katsiadaki, S. R. McCully Phillips, S. Roslyn, M. Sebire, D. Stone, M. J. Witt, S. Wright, N. J. McKeown

**Affiliations:** 1 Centre for Environment, Fisheries and Aquaculture Science, Pakefield Road, NR33 0HT Lowestoft , Suffolk, England; 2 School of Environmental Sciences, University of East Anglia, Norwich, Norfolk, NR4 7TJ UK; 3 Hatherly Laboratories, University of Exeter, Prince of Wales Road, Exeter, Devon, EX4 4PS UK; 4 Centre for Environment, Fisheries and Aquaculture Science, The Nothe, Weymouth, Dorset, DT4 8UB England; 5 Department of Life Sciences, Aberystwyth University, Aberystwyth, SY23 3FL UK

**Keywords:** Genotyping and haplotyping, Ecological genetics

## Abstract

In fisheries, genetic based assignment of individuals to their population of origin can benefit efforts aimed at monitoring and managing stocks. Assignment combined with knowledge of the migration history of individuals can provide powerful insights into mechanisms of genetic mixing, for which refined sampling methods are required to minimise any impacts. In this study we tested two minimally invasive swabbing techniques for sampling DNA when attaching electronic satellite tags to Atlantic bluefin tuna (*Thunnus thynnus*) for migration studies. First, DNA was sampled by skin swabbing (hereafter skin swabs) individuals from which there were corresponding fin clip samples. Second, swabs were taken from the applicator poles used to attach electronic tags (hereafter pole swabs). Quantification of DNA from the different sources revealed decreasing yields moving from fin clips, to skin swabs, to pole swabs. The utility of the DNA obtained by both swabbing methods for individual genotyping was then assessed by sequencing of the mtDNA control region and genotyping of six microsatellite loci. In all cases successful genotyping was achieved. For mtDNA an 868 bp fragment was successfully amplified in all samples with 775 bp aligned across individuals revealing 26 haplotypes (overall haplotype diversity = 0.987). All six microsatellites were successfully amplified including a largest allele size of 291 bp. mtDNA and microsatellite genotypes for the skin swabs matched with the corresponding fin clip samples. Although no tissue replicates were available for the pole swab samples the genotypes obtained were unambiguous, consistent across repeated PCRs, and reported no evidence of PCR issues such as large allele drop out. Overall, the genetic data suggested high variability among individuals sampled, comparable to levels of genetic diversity seen within the species’ Atlantic range. The study demonstrates that non-invasive sampling can be used to obtain DNA for population assignment studies and that valuable material can be sampled from tagging equipment.

## Introduction

The Atlantic bluefin tuna (*Thunnus thynnus*; hereafter BFT) is a highly migratory species that is widely distributed across the Atlantic Ocean. The species’ high commercial value has motivated intense exploitation, contributing to pronounced declines in abundance leading to concern about population resilience^1^. There are three currently recognised BFT spawning grounds – the Gulf of Mexico^2^, the slope sea^3^ and the Mediterranean Sea^4^. Tagged tuna show strong fidelity to spawning areas^2^ with genetic^5,6^ and otolith chemistry^7^ data confirming high levels of natal philopatry and reproductive isolation between these spawning groups.

While there have been recent improvements in BFT stock status linked to more restrictive quotas and management regulations^8^, uncertainties remain within the stock assessment^8–10^). ICCAT currently manages BFT as two unmixed stocks delineated into eastern and western components, separated by the 45^o^W meridian. However, genetic studies have confirmed extensive mixing between both stocks in feeding aggregations throughout the Atlantic^5,6^. These studies have also identified subsets of genetic markers that permit assignment of individuals to their natal stocks.

Collecting DNA samples for population assignment is typically done invasively, using fin clips or tissue samples taken from living or harvested fish. Removal of tissue from harvested fish is not usually problematic and occurs in ongoing monitoring programmes. However, the removal of fin tissue from live specimens, even though non-destructive, may still affect behaviour or survival due to stress, injury or post-sampling infection^11–13^. Skin swabbing, by contrast, enables non-invasive sampling of DNA, based on the principle that cellular material and DNA of the sampled individual will rub away from the skin under light pressure. However, it has previously only been undertaken in laboratory studies on other fish species^13,14^. Operationalising such methods for sampling DNA in the field would, alongside developing genomic resources, offer a valuable approach to assessing spatial/temporal mixing patterns and inform tailored management strategies for BFT (and other fish) stocks and/or geographical areas in real time. Even though the quantities of DNA that are collected this way are likely very small compared to those within tissue samples, modern DNA extraction and amplification techniques are capable of providing viable DNA from small samples that can be used to construct genotypes and population demographics^15^.

For the present study, we identified an opportunity to test the effectiveness of two minimally invasive methods to sample DNA for genetic analysis in BFT during ongoing fieldwork. Firstly, we assessed the potential of skin swabbing during fieldwork that required BFT to be tagged aboard fishing vessels. Second, we investigated if DNA could also be obtained from swabbing of tag applicator poles that had been used to attach satellite tags to individual BFT that were not removed from the water. For both types of samples, the quality of extracted DNA was assessed by DNA quantification and then genotyping by means of mtDNA sequencing and fragment analysis of six microsatellite loci.

## Results

Table 1 contains details of all samples. The nature of the sampling meant that two individuals (21272742 and 21272758) each had three corresponding swab sample duplicates. Of the remaining 17 individuals, 11 had two swab duplicates each, with a single swab duplicate for each of the remaining six. Skin swab samples for which there were no corresponding fin clip samples were also obtained for two individuals (21272750 and 21272769). Yields of DNA (in ng/µl) from fin clips were highest (263, *n* = 19 samples, Tables 1 and 2), followed by skin swabs (112.8, *n* = 37 samples), while yields from pole swabs were the lowest at around 5% of that from fin clips (9.6, *n* = 10). Spectroscopic absorbance ratios (A260 nanometres (nm)/A280 nm) indicated that the DNA from fin clips and skin swabs was high quality (mean value = 2 and 1.99 respectively, Tables 1 and 2), while that from pole swabs was of lower quality (mean value = 1.39). Both yield and absorption ratio differed significantly between sampling technqiues (Kruskal-Wallis rank sum tests with two degrees of freedom, χ2 = 32.5, *p* < 0.01 and χ2 = 23.1, *p* < 0.01 for yield and absorption ratio respectively).

Successful mtDNA and microsatellite PCRs were obtained for all samples and yielded unambiguous genotypes. In the case of mtDNA, following sequencing a stretch of 752 bp was aligned across all individuals, whereas for microsatellites genotypes were obtained for all six loci. All cases for which fin clip – skin swab duplicates could be compared revealed complete congruence i.e. mtDNA and microsatellite genotypes obtained for the skin swab samples matched to the genotypes obtained from the corresponding fin sample and results were consistent across skin swab samples where available. Successful PCR and genotypes were also obtained for the pole swab samples. Although there were no duplicate samples for comparison with the pole swabs these samples yielded clear mtDNA sequences and microsatellite genotypes which were consistent across repeated PCRs (3 per sample) with allele sizes corresponding to expectations based on the tandem repeat. Table 3 compares the basic descriptive statistics for the microsatellite for the finclip/skin swab (*n* = 21 individuals) and pole swab (*n* = 10 individuals), and also the microsatellite allele size ranges, showing successful amplification of alleles ~ 290 bp for locus Tth 207 for both types of swab samples. Standardising for sample size using allelic richness revealed similar values between both groups. The microsatellites, the majority of locus - sample comparisons reported non-significant F_IS_ values.

**Fig. 1:**
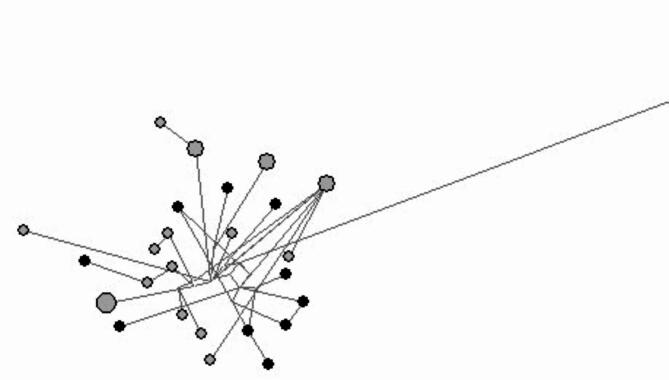
Phylogenetic network showing the relationships among the 26 distinct haplotypes resolved. Discs are proportional to overall abundance and colours to DNA source (grey = skin swab; black = pole swab). Lines connecting discs are proportional to genetic distance and highlight the highly divergent Pacific bluefin tuna haplotype. Fin-clips are not shown, since these were matched to skin swabs in all cases.

Across all 31 individuals genotyped the mean number of alleles per locus was nine, and the mean observed and expected heterozygosities were 0.759 and 0.765, respectively. The overall F_*IS*_ value was 0.047 (NS). Following trimming of mtDNA, a stretch of 752 bp was aligned across the 31 individuals revealing 26 haplotypes (haplotype diversity = 0.987). Sixteen haplotypes were found among the skin swab (h = 0.97) and 10 among the pole swab (h = 1). Phylogenetic reconstruction revealed one sequence (21272770) to be highly distinct (Fig. 1). BLAST analysis of this reported highest similarity to Pacific bluefin tuna (*T. orientalis*).

## Discussion

The improvement and refinement of tissue sampling protocols used on live animals reduces welfare impacts and is an ethical requirement. However, validating the efficacy and reliability of modified sampling protocols is necessary before moving on from the original techniques. Here, we have shown that two non-invasive sampling techniques (swabbing of fish skin and tag applicators) are effective at harvesting DNA suitable for downstream genotyping. This has important implications for studies requiring DNA that previously relied on invasive methods.

In the present study, sampling was carried out with regard to minimising stress where possible, the process of tissue sampling was rapid (< 15 s) and the fin clip sample was small (~ 1 cm^2^) relative to the overall size of the BFT (average length ~ 197 cm). Fin clips are typically taken from fish to use in genetic studies because it is easier to use fin tissue than to take a muscle sample or biopsy^16^. However, the potential impact of fin-clip sampling due to stress, injury or post-sampling infection is not known (but has generally been assumed to be small^16^. Fins are living tissue and are involved in a number of physical and behavioural functions, and are the dynamic driving and sensory surfaces of the fish^17^ and therefore removing any fin tissue should probably be avoided if possible. In contrast, skin swabbing does not require any tissue to be removed, breach of integument, or any blades to be used, and is therefore as quick but safer to perform than a fin clip. In our study, since the BFT was on deck on a tagging mattress during the tagging and sampling process, and because the skin surface of a tuna is very large, the impact of contact with a skin swab is likely of negligible additional impact during the tagging process. Nevertheless, and more generally, Tilley et al.^13^ note that while skin swabs are simpler to perform than fin clips, care must be taken to swab the fish from anterior to posterior using very light pressure to avoid activating nociceptors, which is good practice regardless of fish size. The swab samples taken from tag applicators did not have any additional sampling impact on the subjects beyond the act of tagging and was therefore the most refined protocol.

The quantities of DNA that were collected varied between sampling methods, but in every case were sufficient for downstream PCR amplification and genotyping. The successful amplification of the mtDNA in the samples was not surprising as mtDNA has often been shown to be more readily amplifiable than nuclear loci owing to its higher copy number in cells. In contrast the lower copy number of nuclear loci means that PCR based analysis of low concentration DNA sources may be more prone to stochastic effects such as locus or allele drop out, and is often caused by stochastic sampling of low quality/quantity template DNA obtained from low concentration DNA sources, such as from hair or faeces^18–20^. Although this was a risk with the non-invasive sampling methods we used, we did not observe any locus drop out and all 6 loci were successfully amplified, as evidenced by the comparison between the fin clip and skin swab samples, where there was no evidence of such drop out. Furthermore, although there were no replicates available for the pole swab samples these samples reported no heterozygote deficits that could be indicative of allelic drop out and genotypes were consistent across repeated PCRs from the same extractions. The length and quality of DNA fragments achieved from all samples therefore enabled genotyping, and provided a clear demonstration that the swabbing techniques are suitable for operational use in population genetic studies.

Analysis of low copy number DNA does, however, increase the risk of contamination, which we tested by comparing the variability in haplotypes. One individual was a clear outlier, which can be attributed to a sequence that assigned to Pacific Bluefin Tuna (PBT). Other studies have also reported BFT to yield mtDNA sequences belonging to PBT^21^ as well as albacore tuna (*Thunnus alalunga*^22^). The detection of this PBT sequence can thus be attributed to the established retention of ancestral polymorphism rather than sample contamination or species misidentification.

Across the 31 individuals analysed, levels of nuclear variation and general conformance to Hardy-Weinberg equilibrium were similar to those reported in other microsatellite analyses for the species^22–24^. mtDNA has an effective population that is one quarter that of nuclear loci making it more susceptible to loss of genetic variation. However, levels of mtDNA variation were high (*h* = 0.987) and similar to levels in studies over wider geographical areas. For example, in their study spanning the Mediterranean, Carlsson et al.^22^ reported an *h* of 0.991, while a later study including samples from the east and west spawning areas reported haplotype diversities ranging from 0.949 to 0.997^25,26^. Despite the relatively small sample size in our study, the comparable levels of variation to those reported over the entire species’ range and the detection of the PBT lineage collectively indicate that the BFT population in UK waters has a high level of genetic variability.

Recent developments in genomic methods have highlighted the utility of applying panels of Single Nucleotide Polymorphisms (SNPs) conferring high population assignment power as tools for, real-time regulation of harvesting^27^, cost-effective fisheries enforcement^28^ and alignment of management units with biological patterns of recruitment^29^. Previous studies by Puncher et al.^6^ and Rodriquez-Ezpeleta et al.^5^ have provided valuable resources with regards to developing such SNP panels in BFT. The robust amplification of nuclear loci fragments of > 200 bp and mtDNA fragments over 800 bp highlights that DNA obtained from both swabbing methods could be used in such SNP based analyses.

The use of skin swabbing in the field enables sampling with minimal impacts for study animals. By reducing the impact of sampling on the individual, the probability that stress, injury or post-sampling infection will affect the behaviour, physiology or mortality of the experimental subject should be reduced. Likewise, the harvesting of DNA from tag applicator tools also offers a route for further refining sampling during tagging and genetic studies, and reduces the handling time by eliminating the need for an additional sampling step. Tagging studies have already provided considerable insight into movements patterns of tuna^2,4^. Analysing such patterns alongside population genetic structure could provide considerable insight into the roles of plasticity, genetics, and local adaptation in shaping such patterns^30–32^.

Skin swabbing is a simple technique and, as such, could be adapted by a wider range of practitioners without the need for extensive training or licensing (skin swabbing is not currently considered to be a regulated procedure under the Animals (Scientific Procedures) Act 1986 in the UK). For example, there is considerable potential to use the technique in citizen science projects (e.g. recreational angling studies) and, provided that practitioners are fully trained in the techniques of taking and storing samples, this approach would enable cost-effective sampling programmes that offer wider geographic coverage, over longer time periods. The technique is likely to be effective on many more fish species, but this requires further investigation (e.g. elasmobranchs have armoured skin, so the technique may not be as effective for these species).

## Conclusions

Our data confirmed that both skin swabbing and swabbing of equipment that has come into close contact with BFT provides viable alternatives to traditional invasive approaches (e.g. for sourcing DNA for individual genotyping). As such, both skin swabbing and tag applicator swabbing offer the potential, if part of a tagging programme, to refine handling and genetic sampling techniques, by likely reducing stress and the potential for post-procedural impacts. The SNP genotyping method demonstrated that the refined sampling technique does not compromise the ability to derive sequence data that can be used to assess population of origin or genetic variability in BFT. The wider trialling and use of this non-invasive method is recommended to others engaged in similar studies.

## Methods

**Fig. 2:**
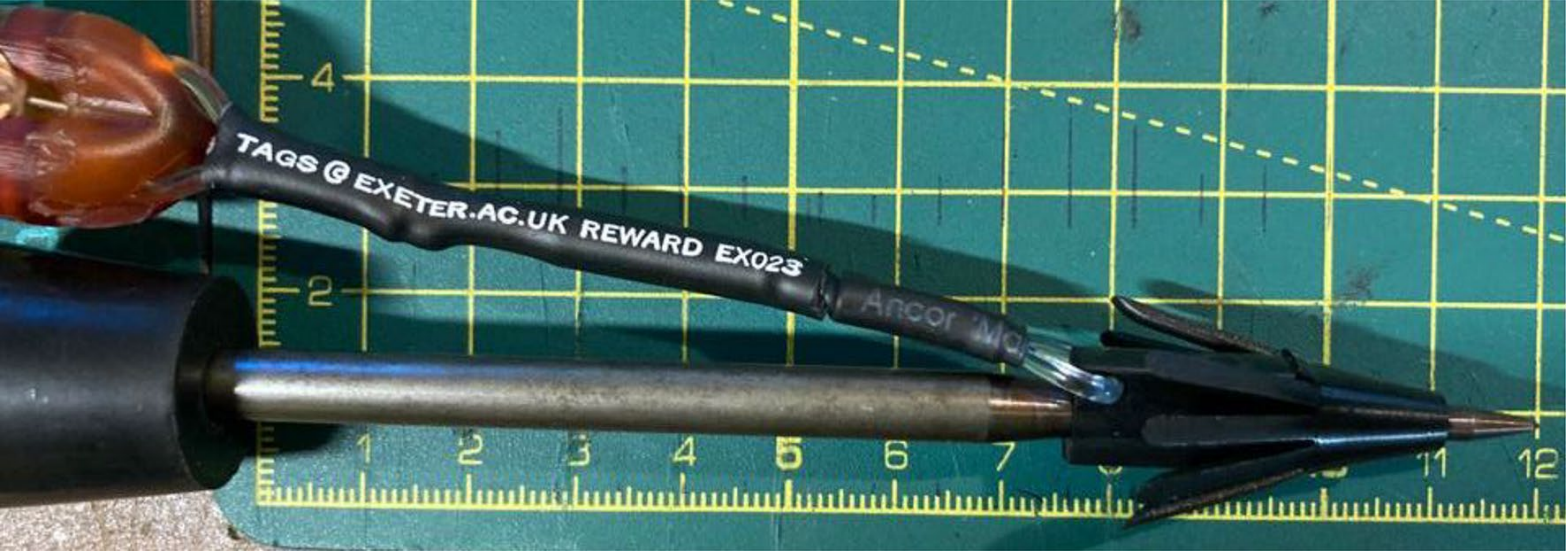
An example of the metal tag applicator used to deploy pop-up satellite tags (PSAT) on BFT. Swabs of the applicator and the stopper bung were taken after tagging to sample the DNA of the tagged individual.

Skin swabs (of approximately 10 cm by 10 cm area, typically in duplicate) and small (approximately 1 × 1 cm) fin clips (taken from the pectoral fin) were sampled from 19 individual BFT (Table 1) that had been caught and brought aboard a charter recreational fishing vessel for the purposes of tagging (electronic pop-up satellite archival tag, or PSAT). For a description of the capture, handling and tagging protocol, see^30,32^. In a separate experiment, swabs (*n* = 10) were taken from tag applicators that had been used to tag BFT with PSAT at the side of fishing vessels (Fig. 2). In this second experiment, no matching fin clips were taken. The tag applicators were cleansed (using iodine wipes) and rinsed copiously with seawater before and after use. Details of all samples are provided in Table 1.

Fin clips and swab tips were immediately stored in microtubes containing absolute ethanol and held in a domestic refrigerator until they could be transferred to -20°C within 12 to 14 hours pending the extraction of DNA. To extract DNA, mucous and cell material was collected by centrifugation at 3,000 g for 30 minutes. The pellet and fin clips were then digested at 56°C overnight in ALT buffer containing proteinase K (Qiagen). The digest was clarified by further centrifugation at 9,500 g for 2 minutes, then the DNA was extracted using the QIAamp 96 DNA QIAcube HT Kit and the Qiacube HT biorobot (Qiagen) and eluted in a 200 µl volume following the manufacturer’s instructions. Polymerase Chain Reaction (PCR) was then used to amplify fragments of the mtDNA and nuclear genomes. mtDNA PCR primers followed Carlsson et al.^22^ wherein a 868 bp fragment of the control region was amplified using the Pro-5’ and 12Sar-3’ primers designed by Palumbi^33^. Sequencing of amplicons was performed using the internal primer (5’-CCATCTTAACATCTTCAGTG-3’)^22^ and BigDye technology. PCR of nuclear markers comprised five dinucleotide loci (Tth1-31, Tth 204, Tth 207, Tth 217; Tth 226^24^) and one tetranucleotide locus (Tth 38^23^). In all cases, PCR mixes comprised 5ul BIOMX (Bioline), 1.0 pMol of primer (both forward and reverse) and 3ul of genomic DNA. PCR thermoprofiles were the same as in the original studies using the primers however, the number of cycles was increased to 55 to mitigate against low copy number DNA. Sequencing and microsatellite products were visualised using ABI 3730 DNA analyser (Applied Biosystems). Sequences were edited using Chromas and aligned using BIOEDIT as per Hall^34^. Haplotype diversity was estimated using DNASP as per Rozas and Rozas^35^ and a haplotype phylogeny was inferred using the software NETWORK V10 (Fluxus Engineering, Free Phylogenetic Network Software). Microsatellite genotypes were inferred using the PEAKSCANNER V2.0 software (Sanger Sequencing and Fragment Analysis Software | Thermo Fisher Scientific - UK). Summary indices of microsatellite variation were estimated using FSTAT as per Goudet^36^ which was also used to calculate and test the significance of F_IS_.

All methods were performed in accordance with the relevant guidelines and regulations and complies with the Animal Research: Reporting of In Vivo Experiments (ARRIVE) guidelines. The work was carried out under UK Home Office Animals (Scientific Procedures) Act (1986), and approved by the local Animal Welfare and Ethical Review Body (AWERB) committees at the University of Exeter (project licence P23C6EFD2) and at Cefas Lowestoft (project licence P9D31EA7F).

**Table 1:** Details of the samples of DNA obtained from swabbing of Atlantic bluefin tuna skin, tagging tools and from fin clips. Length was measured either as curved fork length (CFL) or straight fork length (SFL). Absorption ratio values above 2 are shown in bold to indicate the higher quality of material in these samples.

Sample number	Fish ID	Capture Date	Length type	Length (cm)	Type	Replicate	Yield of DNA (ng/ul)	Absorption at 260 nm	Absorption at 280 nm	Absorption ratio
1	21,272,742	12/09/21	CFL	226	Fin	Primary	69.03	1.38	0.71	1.94
1	21,272,742	12/09/21	CFL	226	Skin	Primary	110.63	2.21	1.03	**2.15**
1	21,272,742	12/09/21	CFL	226	Skin	Duplicate	109.06	2.18	1.01	**2.17**
1	21,272,742	12/09/21	CFL	226	Skin	Triplicate	5.36	0.11	0.1	1.1
2	21,272,744	23/09/21	CFL	136	Fin	Primary	90.4	1.81	0.87	**2.08**
2	21,272,744	23/09/21	CFL	136	Skin	Primary	79.53	1.59	0.82	1.94
2	21,272,744	23/09/21	CFL	136	Skin	Duplicate	118.49	2.37	1.11	**2.14**
3	21,272,746	27/08/21	CFL	213	Fin	Primary	362.59	7.25	3.59	**2.02**
3	21,272,746	27/08/21	CFL	213	Skin	Primary	247.7	4.95	2.33	**2.13**
3	21,272,746	27/08/21	CFL	213	Skin	Duplicate	85.23	1.71	0.85	**2.01**
4	21,272,747	11/09/21	CFL	228	Fin	Primary	149.53	2.99	1.49	**2.01**
4	21,272,747	11/09/21	CFL	228	Skin	Primary	158.75	3.18	1.47	**2.16**
5	21,272,748	29/08/21	CFL	197	Fin	Primary	177.95	3.56	1.85	1.92
5	21,272,748	29/08/21	CFL	197	Skin	Primary	343.79	6.88	3.2	**2.15**
5	21,272,748	29/08/21	CFL	197	Skin	Duplicate	91.17	1.82	0.9	**2.03**
6	21,272,749	28/08/21	CFL	243	Fin	Primary	1371.71	27.43	13.33	**2.06**
6	21,272,749	28/08/21	CFL	243	Skin	Primary	184.83	3.7	1.7	**2.17**
6	21,272,749	28/08/21	CFL	243	Skin	Duplicate	33.24	0.67	0.34	1.93
7	21,272,750	09/09/21	CFL	212	Skin	Primary	52.1	1.04	0.5	**2.11**
8	21,272,754	15/09/21	CFL	208	Fin	Primary	371.71	7.43	3.69	**2.01**
8	21,272,754	15/09/21	CFL	208	Skin	Primary	59.91	1.2	0.58	**2.07**
8	21,272,754	15/09/21	CFL	208	Skin	Duplicate	80.79	1.62	0.77	**2.11**
9	21,272,755	28/08/21	CFL	213	Fin	Primary	265.87	5.32	2.59	**2.05**
9	21,272,755	28/08/21	CFL	213	Skin	Primary	36.46	0.73	0.36	**2.04**
9	21,272,755	28/08/21	CFL	213	Skin	Duplicate	141.67	2.83	1.3	**2.18**
10	21,272,756	21/08/21	CFL	160	Fin	Primary	160.32	3.21	1.55	**2.07**
10	21,272,756	21/08/21	CFL	160	Skin	Primary	94.98	1.9	0.89	**2.13**
11	21,272,758	22/09/21	CFL	150	Fin	Primary	184.05	3.68	1.8	**2.04**
11	21,272,758	22/09/21	CFL	150	Skin	Primary	123.04	2.46	1.13	**2.17**
11	21,272,758	22/09/21	CFL	150	Skin	Duplicate	58.77	1.18	0.54	**2.19**
11	21,272,758	22/09/21	CFL	150	Skin	Triplicate	61.38	1.23	0.62	1.99
12	21,272,760	13/09/21	CFL	229	Fin	Primary	242.28	4.85	2.41	**2.01**
12	21,272,760	13/09/21	CFL	229	Skin	Primary	35	0.7	0.48	1.45
12	21,272,760	13/09/21	CFL	229	Skin	Duplicate	30.52	0.61	0.37	1.66
13	21,272,762	27/08/21	CFL	177	Fin	Primary	300.73	6.02	2.98	**2.02**
13	21,272,762	27/08/21	CFL	177	Skin	Primary	214.04	4.28	2	**2.14**
13	21,272,762	27/08/21	CFL	177	Skin	Duplicate	156.41	3.13	1.46	**2.15**
14	21,272,765	23/09/21	CFL	136	Fin	Primary	176.54	3.53	1.73	**2.04**
14	21,272,765	23/09/21	CFL	136	Skin	Primary	57.1	1.14	0.55	**2.06**
14	21,272,765	23/09/21	CFL	136	Skin	Duplicate	112.87	2.26	1	**2.25**
15	21,272,766	29/08/21	CFL	201	Fin	Primary	194.33	3.89	1.94	**2.01**
15	21,272,766	29/08/21	CFL	201	Skin	Primary	305.77	6.12	2.99	**2.05**
15	21,272,766	29/08/21	CFL	201	Skin	Duplicate	108.72	2.17	1.09	2
16	21,272,769	14/09/21	CFL	207	Skin	Primary	45.77	0.92	0.43	2.13
16	21,272,769	14/09/21	CFL	207	Skin	Duplicate	68.82	1.38	1.18	1.16
17	21,272,770	29/08/21	CFL	207	Fin	primary	358.95	7.18	3.72	1.93
17	21,272,770	29/08/21	CFL	207	Skin	Primary	314.64	6.29	2.96	2.12
17	21,272,770	29/08/21	CFL	207	Skin	Duplicate	59.09	1.18	0.57	2.07
18	21,272,771	27/08/21	CFL	205	Fin	Primary	172.68	3.45	1.7	2.03
18	21,272,771	27/08/21	CFL	205	Skin	Primary	184.57	3.69	1.89	1.95
19	20P0091	14/10/21	SFL	165	Pole	Primary	6.4	0.13	0.1	1.32
20	20P0099	11/10/21	SFL	224	Pole	Primary	6.02	0.12	0.09	1.4
21	20P1107	12/10/21	SFL	216	Pole	Primary	3.44	0.07	0.07	0.93
22	20P1567	16/10/21	SFL	198	Pole	Primary	12.33	0.25	0.13	1.9
23	20P2924	12/10/21	SFL	190	Pole	Primary	4.36	0.09	0.06	1.35
24	20P2936	12/10/21	SFL	183	Pole	Primary	24.96	0.5	0.38	1.32
25	20P2939	11/10/21	SFL	178	Pole	Primary	6.85	0.14	0.09	1.51
26	20P2944	03/11/21	SFL	165	Pole	Primary	16.02	0.32	0.16	1.95
27	20P2945	12/10/21	SFL	191	Pole	Primary	5.14	0.1	0.07	1.41
28	20P2946	11/10/21	SFL	168	Pole	Primary	10.46	0.21	0.25	0.83
29	21P0337	12/10/21	CFL	190	Fin	Primary	91.13	1.82	0.95	1.93
29	21P0337	12/10/21	CFL	190	Skin	Primary	115.67	2.31	1.12	2.07
30	21P0342	12/10/21	CFL	211	Fin	Primary	201.48	4.03	1.97	2.05
30	21P0342	12/10/21	CFL	211	Skin	Primary	60.71	1.21	0.6	2.04
31	21P0399	12/10/21	CFL	201	Fin	Primary	50.32	1.01	0.54	1.87
31	21P0399	12/10/21	CFL	201	Skin	Primary	26.57	0.53	0.4	1.34
NA	Blank	-	-	-	Water	-	1.53	0.03	0.03	0.97

**Table 2:** Summary of DNA extraction and quality assessment from each sample type. DNA yield and quality (using absorption ratio as a proxy) varied significantly with sampling method (Kruskal-Wallis rank sum tests with two degrees of freedom, χ^2^ = 32.5, *p* < 0.01 and χ^2^ = 23.1, *p* < 0.01 for yield and absorption ratio respectively).

Sample type (*n*)	yield (ng/ ml)	260/280 nm absorbtion ratio	Absorption ratio range
Fin clip (19)	262.7 ± 285.3	2 ± 0.06	1.87–2.08
Skin swab (37)	112.8 ± 83.3	1.99 ± 0.28	1.1–2.25
Pole swab (10)	9.60 ± 6.7	1.39 ± 0.35	0.83–1.95

**Table 3:** Summary indices of microsatellite variation for the 6 loci analysed for individuals sampled using skin swab and Pole swabs. These include allele number (na), and observed and expected heterozygosities (HO and HE, respectively). For the skin swab samples allelic richness (ar) is calculated for a sample of *n* = 10 for comparisons with Pole swab. FIS values are used as a measure of conformance to hardy-Weinberg equilibrium within significant deviations denoted by * and assessed using 10,000 permutations.

		Locus					
	Index	**Tth1-31**	**Tth 38**	**Tth 207**	**Tth 226**	**Tth 217**	**Tth 204**
Skin swab (*n* = 21)	N*a* (Ar) *H*_*E*_ *H*_*O*_ *F*_*IS*_ Allele size range	12 (9.4) 0.866 0.895 -0.034 110–144	8 (6.4) 0.791 0.632 0.206* 194–226	5 (4) 0.714 0.684 0.043 281–291	9 (8.1) 0.885 0.842 0.050 158–180	9 (7.6) 0.846 0.842 0.005 237–267	6 (4.8) 0.713 0.789 -0.111 171–183
Pole swab (*N* = 10)	N*a* *H*_*E*_ *H*_*O*_ *F*_*IS*_ Allele size range	10 0.879 0.8 0.094 110–150	5 0.716 0.6 0.169 194–210	3 0.674 0.9 -0.361* 281–289	6 0.863 0.6 -0.095 158–180	8 0.826 0.9 -0.095 239–261	4 0.753 0.5 0.348 171–181

## Data Availability

All sequences are available on GenBank with accession numbers OR625590-OR625653.
